# Barriers and Facilitators to COVID-19 Vaccine Uptake among Polish Patients: A Qualitative Interview Study

**DOI:** 10.3390/vaccines11010177

**Published:** 2023-01-13

**Authors:** Ludmiła Marcinowicz, Ewa Fejfer-Wirbal, Agnieszka Dudzik, Agnieszka Genowska, Sławomir Terlikowski

**Affiliations:** 1 Department of Obstetrics, Gynecology and Maternity Care, Medical University of Bialystok, 15-295 Bialystok, Poland; 2 Department of Health Sciences, Prof. Edward F. Szczepanik State Vocational University in Suwalki, Noniewicza 10, 16-400 Suwalki, Poland; 3 Department of Foreign Languages, Medical University of Bialystok, 15-222 Bialystok, Poland; 4 Department of Public Health, Medical University of Bialystok, 15-295 Bialystok, Poland

**Keywords:** COVID-19, vaccine uptake, vaccination barriers, Polish patients, qualitative research

## Abstract

The decision to receive a COVID-19 vaccine is influenced by a variety of individual and contextual factors. However, there are very few studies that analyse individual vaccination decisions using a qualitative methodology. To close this gap, we conducted a qualitative interview study to examine the opinions and experiences with the vaccine among patients previously hospitalized due to COVID-19, including barriers and facilitators to vaccine uptake. An exploratory qualitative study, using semi-structured telephone interviews, was conducted among 22 patients admitted for COVID-19 in Poland in 2022. Opinions of patients previously hospitalized with COVID-19 on vaccination were varied. Barriers to COVID-19 vaccine uptake stemmed from concerns about vaccine safety, patients’ religious beliefs, and negative stories. High disease severity and anxiety over personal and family health were important arguments in favour of receiving the COVID-19 vaccine. The study findings indicated the need for ongoing health education by healthcare staff as well as coordination and integration of multi-sectoral institutional measures regarding COVID-19 prevention strategies as well as increased public health initiatives on social media and engagement of community leaders for awareness about vaccines and vaccination. It is crucial to build trust in COVID-19 vaccinations among the general public by disseminating reliable information through trustworthy and credible sources. However, it ought to be emphasised that, regardless of the measures taken, some individuals will remain unconvinced about receiving a COVID-19 vaccine.

## 1. Introduction

The decision to get vaccinated against COVID-19 depends on a variety of individual and contextual factors. Despite widespread recommendations to vaccinate, a considerable level of COVID-19 vaccine hesitancy is still being observed [1]. The underlying reasons for vaccine reluctance are complex and context-dependent, i.e., with regard to time, place, and vaccine availability [2]. Various initiatives are being adopted across Europe to enhance COVID-19 vaccination coverage and to vaccinate all eligible persons [3]. In Poland, a national lottery with material rewards and cash prizes for those fully vaccinated was launched in July 2021 [4]. Polish municipalities also competed to achieve the highest vaccination rates.

A review of the literature indicates great differences in COVID-19 vaccination rates across many countries, including Poland. The results of surveys conducted in 2020 in 19 countries revealed that 56.31% of the population surveyed in Poland would take a COVID-19 vaccine, provided that a safe and effective one was available. Significantly, differences in acceptance rates ranged from 88.62% in China to 54.85% in Russia [5]. Surveys conducted in 32 countries (including Poland) to determine acceptance of a COVID-19 vaccine showed that intentions to receive it were low in Lebanon, France, Croatia, and Serbia, with polarisation in acceptance rates across Poland and Pakistan. Moreover, Poland was one of the five countries (alongside Croatia, France, Lebanon, and Paraguay) with a higher proportion of respondents who stated that they would ‘definitely not’ take the vaccine than would ‘definitely’ take it (21.0% and 19.6%, respectively) [6]. A cross-sectional survey of 1071 adults in Bosnia and Herzegovina in early 2021 indicated that only 25.7 per cent of respondents were interested in receiving a COVID-19 vaccine. Age, education, and income factors all improved vaccination uptake and acceptance. The main predictors of vaccination uptake were the desire for herd immunity and concern for one’s own health. In contrast, the majority of arguments against vaccinations stemmed from a lack of adequate clinical trials [7]. Other researchers used medical records to investigate motivations and concerns about COVID-19 vaccination. The study included 102 individuals who sought medical advice prior to receiving a vaccination. Protection against COVID-19, employment obligation or requirement, medical advice from a primary healthcare provider, personal decision to vaccinate, and civic duty were all indicated as reasons for getting vaccinated [8].

There are several factors associated with the disparities in COVID-19 vaccination coverage, including the dynamics of supply and service provision in healthcare systems and individuals’ beliefs, attitudes and behaviours. Underlying issues, which can all combine to create conditions where vaccination uptake is lower than desired, include, although are not limited to, the following: distrust of government; disease risk perception; prior experiences, such as a fear of vaccines; uncertainties about vaccine safety and effectiveness; and evolving policy decisions regarding pandemic management [3].

Data from mid-August 2022 show that 61.9% of the world’s population has been vaccinated against COVID-19. The share of people with a complete initial protocol varies by continent. It ranges from 83.89% in Australia (8 November 2022) to 21.43% in Africa, while reaching 66.04% of the population in Europe. Poland, with 58.83% (12 August 2022) of the population having been vaccinated, is among the countries with the lowest vaccination rates in Europe [9].

Determining the effectiveness of an intervention aimed at facilitating vaccination uptake is a methodological challenge. An effective COVID-19 vaccination programme can only be built on understanding and appropriate responses to the beliefs, concerns, and expectations of individuals and communities. It should also be designed to address their perceptions and concerns regarding COVID-19 [3]. Understanding the barriers to COVID-19 vaccine uptake can help in designing better-targeted interventions. It is important to emphasise that hesitancy to receive the COVID-19 vaccine is a global problem that requires monitoring. This can play a crucial role in reducing the subsequent healthcare burden of COVID-19. Evidence on reasons related to the decision to (not) vaccinate is widely discussed in the literature and continues to grow, but it is mainly based on quantitative studies [10,11,12,13]. Very few studies have used qualitative methodologies to analyse individual COVID-19 vaccination decisions. Significantly, qualitative findings can help to explain people’s attitudes and behaviours when making vaccination decisions. A recent qualitative study conducted in the USA highlighted that vaccine decisions build from the complexities of individuals’ experiences and cultures [14]. It also indicated that the main factors associated with vaccine hesitancy were mistrust, fear, and lack of information [15]. Several studies performed in Europe have indicated that a perceived lack of need for vaccination, concerns about vaccine safety and effectiveness, and accessibility issues act as barriers to vaccine uptake [16]. However, it seems that no research investigating patients’ views on vaccination following hospitalization due to COVID-19 has been published to date. To close this gap, we performed a qualitative interview study to examine the views and experiences with the vaccine among patients previously hospitalized due to COVID-19, including barriers and facilitators to vaccine uptake.

## 2. Materials and Methods

### 2.1. Design

An exploratory qualitative study [17] using semi-structured telephone interviews was conducted among patients previously hospitalized with COVID-19 in Poland. The interviews were conducted between January and May 2022. The length of interviews ranged from 10 to 26 min.

Purposeful sampling was used to recruit patients from a range of demographics and with a range of different hospitalization experiences. The interviews were conducted until unique categories were no longer identified and data saturation was achieved. Methodological issues and limitations of saturation in qualitative research were considered [18].

The study was approved by the Bioethics Committee of the Medical University of Bialystok, Poland (No. APK.002.513.2021).

### 2.2. Participants

Only adults (aged 18 years and over) were invited to participate in the study. All respondents were patients of primary health care facilities in the Podlaskie Voivodeship and had previously been hospitalized due to COVID-19. Invitations to participate in the study were administered via e-mail or telephone. Two female patients refused to take part in the interview, despite the researcher’s assurances that no personal data would be disclosed, and that the study was for research purposes only. The patients stated that they did not wish to disclose their attitudes towards COVID-19 vaccination.

### 2.3. Measurements

The research tool was an interview guide that was developed on the basis of the existing literature and previous findings of researchers investigating COVID-19. The interview was guided by the following questions:

- What influenced your decision to get vaccinated?
- What made your decision to get vaccinated easier?
- Have you had any concerns about vaccination?
- Will you come for a booster immunization?
- Why do you think some people do not want to be vaccinated against COVID-19?
- What (or who) do you think would encourage/persuade people to get vaccinated against COVID-19?

All interviews were conducted by one researcher, were digitally recorded, and were transcribed verbatim by the second author of the paper.

### 2.4. Data Analysis

The transcripts were subjected to thematic analysis. In the process of thematic analysis, the six stages reported by Kiger and Varpio [19] were adopted: becoming familiar with the data, generating initial codes, searching for themes, reviewing themes, defining themes, and writing up. The data were discussed among the research team at each stage of the process. All the authors were involved in the data analysis process.

This article adheres to the Consolidated Criteria for Reporting Qualitative Research (COREQ) guidelines [20].

## 3. Results

The study included 22 patients hospitalized due to COVID-19. The time since discharge from hospital ranged from 3 months to 2 years. The sample was composed of eight females and 14 males. Their mean age was 58.8 years, with the youngest being 38 years old and the oldest one 77 years old. The study group included eight people with higher education, seven with secondary education, four with vocational education, and two with primary education. Nine participants had received a full three-dose vaccination against COVID-19, seven had received partial vaccinations, and five of them were not vaccinated at all. Of the 22 patients, 16 were vaccinated after hospitalization. The characteristics of the study participants are presented in Table 1.

### 3.1. Sources of Information about Vaccinations

Analysis of responses regarding the sources of information about vaccinations revealed the following four categories: 1. the Internet; 2. press and television; 3. conversations with friends; 4. family doctors.

#### 3.1.1. Internet

Most study participants acknowledged the Internet as being an easily accessible and increasingly popular platform that offers numerous possibilities of obtaining various information. Some respondents indicated the Internet as an information source in general terms, without providing any further details, e.g., ‘*There is a lot of data available on the Internet*’ (Interview 2).

Social networking sites disseminating content related to adverse vaccine events also seemed to be a popular source of information. Study participants accessing them were convinced of the reliability of data available on social media: ‘*A Facebook group where users talk about what happened to people following vaccination. Maybe I am very gullible, but I don’t think there is any reason for anyone to lie. Right?*’ (Interview 7).

Other respondents chose to depend on different online sources, as they were convinced of the scientific nature of the data they included: ‘*I get information from science podcasts and news podcasts as well as YouTube’s science channels and news channels*’ (Interview 1).

#### 3.1.2. Press and Television

The data obtained in the study indicate that the information communicated by people recognised as experts, in the press and television, was also considered a reliable source of information about vaccinations: ‘*There is a lot of information in the press. Our celebrities, superstars, doctors, etc. give interviews*’ (Interview 2).

#### 3.1.3. Conversations with Friends

A striking finding to emerge from the study was that the pandemic situation and reports regarding morbidity, hospitalizations, deaths, and vaccinations permeated nearly every aspect of daily life and dominated conversations in a variety of contexts, including the workplace:

‘*I work with a very large number of people and therefore, when discussing this topic, we learn all these different things and that’s where I get most information from*’.

(Interview 2)

Significantly, pandemic-related information was also circulated during meetings with neighbours or family: ‘*Well, we talk about such things with my neighbours, for example, or with my family. For instance, my sister works in a school and she says that some vaccinated people also caught COVID and got severely ill, and one girl even ended up in hospital*’ (Interview 7).

#### 3.1.4. Family Doctors

Some study participants indicated that their GPs provided them with information on vaccination: ‘*From the family doctor. The doctor said it was better to get vaccinated. That was the suggestion she gave me, well, because you don’t know what can happen next*’ (Interview 14).

### 3.2. General Perceptions of COVID-19 Vaccinations

The data obtained from study participants covered a range of issues related to the perceptions of COVID-19 vaccinations. The findings can be divided into the following four sub-themes: (1) trust in vaccine effectiveness, (2) vaccine-associated reduction in symptom severity, (3) trust in scientists, (4) belief in natural immunity.

#### 3.2.1. Trust in Vaccine Effectiveness

A salient point to emerge from the study is that respondents who had received the COVID-19 vaccination were convinced of the need for such vaccines and highlighted their important role in combating the pandemic: ‘*If the society, or humanity in general, does not get vaccinated, we will never control this virus in our lifetime. More and more mutations are being discovered and becoming more and more difficult to control. This vaccination has been shown to be highly protective*’ (Interview 8).

#### 3.2.2. Vaccine-Associated Reduction in Symptom Severity

Significantly, study participants were aware of the fact that they could become reinfected despite being vaccinated. However, they believed that those who had been vaccinated were likely to have a milder course of the disease: ‘*I regretted not taking the time to get vaccinated. I ended up in hospital. If I had been vaccinated in time, maybe the disease course would have been even milder*’ (Interview 12).

Additionally, some unvaccinated respondents believed that COVID-19 vaccinations could have a beneficial effect on the course of the disease: ‘*Some people had been vaccinated and yet they experienced severe symptoms of COVID, one girl was even taken to hospital. This is a bit of a disincentive to vaccination. Yet, I still think that, although she was in hospital, she could have been much worse if she hadn’t taken the vaccination. I think vaccinations don’t guarantee that a person certainly won’t get infected again. You can get reinfected and get sick, but you will have milder symptoms*’ (Interview 7).

#### 3.2.3. Trust in Scientists

Some of the opinions on COVID-19 vaccinations contained references to other recommended vaccinations. Significantly, study participants emphasised their trust in scientists and scientific knowledge: ‘*I just trust people who are involved in science or medicine. If they believe that one should get vaccinated, I simply see no reason not to vaccinate. Even more so because I have been taking the influenza vaccine for the last 6 years and have not had flu once since then*’ (Interview 12).

#### 3.2.4. Belief in Natural Immunity

Some study participants were sceptical about COVID-19 vaccinations. They expressed a preference for developing immunity without the interference of vaccination: ‘*I was against the vaccination mainly because I just always prefer, somehow in a natural way, when my body tries to fight diseases itself*’ (Interview 20).

### 3.3. Barriers to COVID-19 Vaccine Uptake

It is common knowledge that the implementation of COVID-19 vaccination was met with both positive and negative responses. Study participants expressed their views on the reasons against vaccination which, in their opinion, were reported by those refusing to be vaccinated. The reasons for non-vaccination were grouped into the following six sub-categories: (1) vaccine safety concerns, (2) doubts about vaccine effectiveness, (3) health concerns and fear of vaccine complications, (4) religious beliefs and other factors, (5) disinformation and vaccination rumours, and (6) restriction of freedom (Table 2).

#### 3.3.1. Vaccine Safety Concerns

Significantly, concerns about rushed vaccine development were indicated as one of the reasons for limited confidence in its safety: ‘*I don’t know…. If this vaccine had been on the market for 10 years already, there would be no complications from it…. It has been around for too short, there’s not enough information about it*’ (Interview 11).

‘*Everything has been quick. And what effects will the vaccine have in 5–7 years? Will it have any long-term effects on the body? That is an unknown, right?*’.

(Interview 13)

#### 3.3.2. Doubts about Vaccine Effectiveness

Several study participants believed that the COVID-19 vaccine was ineffective. Interestingly, they considered medication to be helpful and believed that immunity acquired by infection is greater than that acquired by vaccination: ‘*People don’t believe in COVID-19 vaccination. My family is also divided over attitudes to vaccination*’ (Interview 19).

‘*There are different things that people here say about it. They say that you sometimes get immune through overcoming natural infection and that it gives you more immunity than the vaccines*’.

(Interview 11)

#### 3.3.3. Health Concerns and Fear of Vaccine Complications

Some study participants indicated underlying medical conditions or symptoms following the previous dose of vaccine as reasons for vaccination refusal: ‘*Because I have experienced anaphylactic shock following the administration of certain drugs. There is no scientific data available on whether I could be allergic or not to a particular substance used in the vaccine. So here I am, taking care of my own health and of my own life. I have not been vaccinated and I would not want to be*’ (Interview 11).

‘*I will state it openly that I will not have my second dose of the vaccine due to the fact that I had severe side effects following the first dose. I had very bad headaches and I had never experienced anything like that before*’.

(Interview 20)

Other respondents had heard of adverse vaccine reactions reported by other people. They considered vaccine-associated adverse events as a barrier to vaccination uptake: ‘*Out of fear for their own health. I mean that people are afraid that the vaccine might unnecessarily “change them”. It might—in inverted commas—mess with their body or change their DNA. Some of them are afraid of post-vaccination complications, such as thrombosis, etc.*’ (Interview 1).

#### 3.3.4. Religious Beliefs and Other Factors

Several study participants emphasised that religious beliefs may influence COVID-19 vaccination decisions: ‘*Faith. I have a friend who did not get vaccinated for religious reasons. In her opinion, what God created is perfect and vaccination is like interfering with that*’ (Interview 18).

One respondent stated that their decision was influenced by their next of kin: ‘*My wife told me not to vaccinate. She had some immunological diseases. Well, and she said that I shouldn’t get vaccinated just like her*’ (Interview 15).

#### 3.3.5. Disinformation and Vaccination Rumours

The data generated in the study revealed that the barriers to COVID-19 vaccine uptake included the wide dissemination of and access to information regarding COVID-19: ‘*People access some inaccurate information and simply become influenced by someone. We all know what’s happening on the Internet, in various reports, different rumours. This mainly scares people. Well, because all the people I meet are simply scared*’ (Interview 16).

#### 3.3.6. Restriction of Freedom

With regard to calls for compulsory vaccination for specific groups, one respondent indicated restriction of freedom as a reason for not receiving a COVID-19 vaccine: ‘*As regards compulsory vaccination for certain groups, I hear the narrative that it is a restriction of freedom. If people want to endanger their own health, that’s their business. But if they threaten others—right?*’ (Interview 21).

### 3.4. Facilitators to COVID-19 Vaccine Uptake

The findings concerning facilitators to COVID-19 vaccine uptake can be divided into the following two categories: (1) determinants of COVID-19 vaccine acceptance and (2) motivating factors for vaccine uptake (Table 3).

#### 3.4.1. Determinants of COVID-19 Vaccine Acceptance

The following five determinants of COVID-19 vaccine uptake were identified in the study: (1) severe course of COVID-19, (2) safety of self and loved ones, (3) severe illness and death in the family due to COVID-19, (4) travel restrictions, and (5) lack of concern about vaccination.

##### Severe Course of COVID-19

Study participants admitted that contracting and suffering severe COVID-19 influenced their decisions regarding vaccination. Some respondents described their hospitalization and witnessing patient suffering and death as traumatising: ‘*If someone hasn’t been in the ward and seen it all, how they carry away—in those bags—the people you had spoken to 15 minutes before…. Four people died during my stay in the ward. My condition wasn’t good either, because more than 70% of my lung tissue was diseased. That experience largely influenced my decision to get vaccinated*’ (Interview 3).

##### Safety of Self and Loved Ones

Some respondents indicated their own safety as a reason for taking the vaccine: ‘*I work with COVID-19 patients and getting vaccinated results in better protection against the disease*’ (Interview 8).

Other study participants highlighted the safety of their loved ones: ‘*The safety of my family. I have children and grandchildren. I was concerned about their safety when they came to see me*’ (Interview 3).

‘*For my husband’s sake. I don’t have any underlying health conditions that increase risk of severe disease associated with COVID-19, but my husband is asthmatic. In fact, I got vaccinated because of him*’.

(Interview 20)

##### Severe Illness and Death in the Family Due to COVID-19

Several respondents stated that the illness of their loved ones or the loss of a family member due to COVID-19 affected their decision to get vaccinated: ‘*In my family, my brother also contracted COVID. He ended up in hospital in a very serious condition and that made me decide to take the vaccine*’ (Interview 13).

‘*What motivated me to get vaccinated was something of a very personal nature, I think. I contracted the disease and I also lost my sister to COVID-19*’.

(Interview 18)

##### Travel Restrictions

Some respondents decided to receive a COVID-19 vaccine for travel purposes. They emphasised that people wishing to travel abroad were required to be fully vaccinated and to provide proof of vaccination status: ‘*Because of the introduction of Covid-19 passports. I was worried about travel restrictions because I travel a lot for work. I got vaccinated in order not to make things more difficult for myself, as they say*’ (Interview 16).

##### Lack of Concern about Vaccination

A striking finding to emerge from the data is that memories of childhood vaccinations had a positive impact on the decision to get vaccinated against COVID-19: ‘*I was vaccinated as a child. Somehow, I have been programmed since my childhood to believe that you should be vaccinated*’ (Interview 17).

In addition, trust in modern medicine and the benefits of vaccination were emphasised: ‘*I knew that COVID-19 could kill me. Vaccination, however, could not kill me. Instead, it could only make me stronger, only that*’ (Interview 8).

#### 3.4.2. Motivating Factors for Vaccine Uptake

Analysis of the study findings revealed the following seven motivating factors for vaccine uptake: (1) incentive programmes, (2) health concerns, (3) belief in vaccine safety, (4) vaccine education, (5) vaccination obligation, (6) uniformity of vaccine information, and (7) voluntary decision.

##### Incentive Programmes

According to study respondents, a variety of incentives, including a day off work or a financial reward, could encourage vaccine uptake: ‘*I am convinced that paid time off for people to be vaccinated would persuade those who are not fully convinced. It is the same with donating blood. If we go to donate blood, we get a day off from our employer*’ (Interview 1).

‘*What would convince a Polish person? Either a legal obligation or money. In my opinion, offering money for vaccination doesn’t seem a humane or ethical thing to do. Various lottery incentives have been tried out, but they have had no effect, as you can see*’.

(Interview 18)

##### Health Concerns

Some respondents felt that a traumatic experience of illness or the loss of a loved one due to COVID-19 could influence the decision regarding vaccination: ‘*My sister, who could also get vaccinated, had chosen not to do it. She had been putting it off all the time. When I got infected, she saw what I had to go through. I got ill and required mechanical ventilation. She got vaccinated the following day*’ (Interview 10).

##### Belief in Vaccine Safety

Being assured of vaccine safety and a lack of adverse reactions following vaccination could convince several respondents to get vaccinated: ‘*If I could know that nothing happens to people who take the vaccination, I would get vaccinated*’ (Interview 7).

##### Vaccine Education

Participating in this study, i.e., being interviewed, could positively influence some individuals who were undecided about vaccination: ‘*Who knows—maybe I will talk to you and think it over again, rethink my attitude*’ (Interview 7).

‘*Definitely vaccine education, in general terms*’.

(Interview 18)

##### Vaccine Obligation

Respondents highlighted the need to introduce mandatory vaccination for certain groups of people: ‘*Some groups of people should literally be forced to get vaccinated. Firstly, healthcare workers, and secondly, teachers. Also, grocery shop staff, as they have direct contact with different customers*’ (Interview 21).

##### Uniformity of Vaccine Information

Some study participants observed that uniform messages to the public could have a positive impact on attitudes towards vaccination: ‘*It would have to be a uniform message from all media, both government and independent. However, that message has not been uniform so far. As a result, we have a divided society and the vaccination issue is ambiguous*’ (Interview 22).

##### Voluntary Decision

Other participants claimed that the decision to get vaccinated must be made by each individual themselves: ‘*It’s everyone’s individual decision. I think everyone has to decide for themselves. No one should be persuaded into doing anything*’ (Interview 9).

## 4. Discussion

This qualitative interview study reported on barriers to and facilitators of vaccine uptake among Polish patients previously hospitalized due to COVID-19. Barriers to vaccination included vaccine safety concerns, doubts about vaccine effectiveness, health concerns and fear of vaccine complications, religious beliefs and other factors, disinformation and vaccination rumours, and restriction of freedom. Factors facilitating respondents’ acceptance of the vaccine included a severe COVID-19 course, safety of self and loved ones, severe illness or death in the family due to COVID-19, travel restrictions, and lack of concern about vaccination.

Opinions on vaccination hesitancy in our qualitative study are in part consistent with other findings, such as the disbelief in vaccine safety and fear of side effects [21,22]. On the other hand, differences across countries and their healthcare systems can be observed [21,23]. Others highlighted their perceived lack of need for vaccination, access difficulties, and treatment issues due to ethnicity as barriers [16,21,24]. A study by Steinert et al. [25] on vaccine hesitancy in eight countries, including Poland, indicated a variety of country-specific reasons for vaccine hesitancy. Female gender and lower education levels were among the most common ones in several countries [25]. Significantly, this is consistent with other recent studies on COVID-19 vaccine hesitancy [26,27,28].

Our study confirms reports by other authors [29] that a lack of accurate information on the one hand and the spread of misinformation on the other are among the most common barriers to COVID-19 vaccine uptake. Data from a British qualitative study by Lockyer et al. indicate that hesitancy to vaccinate is due to safety concerns, negative stories and personal knowledge [22]. Moreover, another study participants indicated perceived lack of need for COVID-19 vaccinations, vaccination efficacy concerns, safety concerns, and access issues as barriers [16]. Significantly, confusion and distrust also reduce positive attitudes towards vaccination. A study by Loomba et al. conducted in the UK and in the USA confirms that misinformation and inadequate knowledge can be barriers to vaccine uptake decisions [30].

In our study, the most common source of information that participants accessed about COVID-19 was the Internet. A study by Fan et al. that evaluated the quality of information on COVID-19 available on websites originating from 34 countries indicated the need for higher quality online resources on the topic to facilitate public education and health measures in the field of public health [31]. The Internet should be used to provide reliable information and current knowledge of vaccination [13]. Polish scientists proposed an action strategy involving organising groups of recognised experts to communicate scientific facts on COVID-19 vaccines to the general public. In this way, reluctance to vaccinate can be reduced [32]. Providing people with reliable and accurate information regarding the prevention and treatment of COVID-19 is essential.

Our study shows that COVID-19 vaccine uptake can be challenged by religious beliefs. The findings are consistent with those observed in earlier research showing that religiosity is negatively correlated with COVID-19 vaccination [33]. A recent study suggested that some religious groups display a lack of trust in science, which accounts for inadequate knowledge related to available vaccines, as vaccine research is based on innovative technologies [34]. In consequence, this lack of knowledge is reflected in greater vaccine hesitancy. Nevertheless, other studies did not confirm a correlation between religion and vaccination readiness [35]. Such inconsistencies may be a result of differences related to religious beliefs and their local socio-cultural context. In addition to religion, other possible reasons for COVID-19 vaccine hesitancy included no vaccine approval by authorizing agencies such as the FDA and the CDC, concern over serious reactions, anxiety related to being jabbed, transportation issues or infrastructure problems, and personal freedom to choose or decide [1]. It needs to be emphasised that vaccine hesitancy and the resultant poor vaccination coverage can contribute to a delay in achieving herd immunity through vaccination.

Health practitioners remain the most trusted source of information on COVID-19 and should therefore address patient safety by providing scientific expertise. Recommendations from medical authorities can increase patients’ trust in the vaccine, thus leading to reduced anxiety about receiving it [36]. However, it is believed that education by health practitioners is more successful when offered in conjunction with public health initiatives. Significantly, coordinated multi-sectoral measures are more effective compared to health interventions involving single institutions. Informed collaboration between institutions, including the government with the Ministry of Health and representatives of other ministries, local health institutions, pharmaceutical companies, and community-based organisations, is the suggested remedy for policies intended to address vaccine hesitancy [37,38]. This approach can include engaging local leaders, community mobilisation, and mass media campaigns, as well as efforts to increase knowledge and awareness about vaccines and vaccination [37]. The knowledge and expertise of independent public health professionals, as well as other resources, should all be utilised as part of a multi-sectoral approach to instil public trust in vaccines [38]. Additionally, research suggests that addressing sensitivity to religious beliefs is an important strategy for increasing vaccine acceptance [39]. Social media, which can provide positive and convincing opinions regarding vaccines, are a crucial instrument for increasing awareness and knowledge about vaccination.

In order to improve vaccination uptake, many researchers emphasise the need to build communication strategies. One of them is to undertake initiatives targeting the general public with the support of recognised researchers [15]. Reaching out to communities with unfavourable vaccination attitudes or low vaccine uptake is a suggested course of action [40,41]. Some experts recommend enhancing medical training for health professionals and providers [36,42]. Vaccination hesitancy among healthcare workers may have an impact on patients’ decreased vaccine uptake. Thus, information must be communicated in a more comprehensible manner and best practices must be followed [43,44]. Caution in the implementation of communication strategies to avoid escalating resistance among non-vaccinated communities is emphasised [14].

Reluctance and hesitation regarding vaccination are not new phenomena [45]. The sudden outbreak of the COVID-19 pandemic and the widespread availability of social media resulted in the rapid spread of misinformation, disinformation and conspiracy theories. Policy decisions taken by some governments were not always in line with reliable data [46]. Consequently, the complexity of the situation was not conducive to COVID-19 vaccine uptake.

### Strengths and Limitations

To the best of our knowledge, our qualitative interview study is the first report showing the results of barriers to and facilitators of vaccine uptake among Polish adult patients previously hospitalized due to COVID-19. Undertaking this type of research is crucial due to the severe socioeconomic burden of the pandemic. It needs to be emphasized that only a few published qualitative studies have shown barriers and facilitators to vaccine uptake in populations from other countries, although they did not include individuals previously hospitalized due to COVID-19 [14,16,21,23,30,47,48]. The greatest value of our study lies in the fact that the respondents were recruited from several hospitals and thus had experiences of hospitalization in several different healthcare institutions. Our study’s greatest strength is that each participant had been previously hospitalized due to COVID-19 and the qualitative approach allowed for a thorough investigation of their opinions and perceptions of the factors that influence vaccine uptake rates. Additionally, the study was conducted when all participants had access to free COVID-19 vaccines provided through a national government programme. Moreover, the researcher conducting the interviews was in no way related to the provision of medical services yet had experience in conducting qualitative research. Furthermore, the process of data analysis was undertaken in an interdisciplinary team and all doubts were resolved through discussion.

This study also possesses several limitations. One of them arises from the purposive sampling resulting from the methodological approach adopted. Despite the fact that thematic analysis is relatively simple to implement, the flexibility it offers is seen as a disadvantage, as it is not a rigorous method and thus has several pitfalls [19]. In our data analysis, an overlap between some sub-themes occurred. Moreover, matching some statements to the subthemes was challenging. An attempt to follow the principles described in the literature was made in order to ensure the trustworthiness of our results and their interpretation [19]. Nevertheless, our qualitative study can be used as a basis for further quantitative research to survey a larger sample of respondents and allow for statistical analysis.

## 5. Conclusions

Our research highlights several barriers to and facilitators of vaccine uptake among Polish patients previously hospitalized with COVID-19. However, key to individual decisions regarding vaccine uptake are factors relating to knowledge, attitudes and religious beliefs, and consciousness of health consequences associated with COVID-19. The study findings indicated the need for ongoing health education by health care personnel as well as coordination and integration of multi-sectoral institutional measures regarding COVID-19 prevention strategies, as well as increased public health campaigns on social media and engagement of community leaders for awareness about vaccines and vaccination. It is important to build confidence in COVID-19 vaccination among the general public by disseminating reliable information through trusted scientific channels. However, it needs to be emphasised that, regardless of the measures taken, some people will remain unconvinced, and will therefore be unwilling to receive the COVID-19 vaccine.

## Figures and Tables

**Table 1:** Participant characteristics (N = 22).

Characteristics	N (%)
Gender	
Female	8 (36.4)
Male	14 (63.6)
Age	
38–40	2 (9.2%)
41–50	3 (13.6%)
51–60	7 (31.8%)
61–70	7 (31.8%)
≥71	3 (13.6%)
Education	
Primary	3 (13.6%)
Technical/Vocational	4 (18.2%)
Secondary	7 (31.8%)
Higher	8 (36.4%)
Time since hospitalization (in months)	
3–6	4 (18.2)
7–12	9 (40.9)
13–18	8 (36.4)
19–24	1 (4.5)
Number of COVID-19 vaccine doses of taken	
1 dose, no intention to take another dose	3 (13.6)
2 doses, intention to take a 3rd dose	4 (18.2)
2 doses, undecided about a 3rd dose	1 (4.5)
3 doses	9 (40.9)
none	5 (22.8)

**Table 2:** Barriers to COVID-19 vaccine uptake.

Category	Sub-Categories
Barriers to COVID-19 vaccine uptake	Vaccine safety concerns
	Doubts about vaccine effectiveness
	Health concerns and fear of vaccine complications
	Religious beliefs and other factors
	Disinformation and vaccination rumours
	Restriction of freedom

**Table 3:** Facilitators to COVID-19 vaccine uptake.

Category	Sub-Categories
Determinants of COVID-19 vaccine acceptance	Severe course of COVID-19
	Safety of self and loved ones
	Severe illness and death in the family due to COVID-19
	Travel restrictions
	Lack of concern about vaccination
Motivating factors for vaccine uptake	Incentive programmes
	Health concerns
	Belief in vaccine safety
	Vaccine education
	Vaccination obligation
	Uniformity of vaccine information
	Voluntary decision

## Data Availability

Due to the sensitive nature of the data, it is available from the corresponding author upon reasonable request.
